# Gait disturbances and muscle dysfunction in fibroblast growth factor 2 knockout mice

**DOI:** 10.1038/s41598-021-90565-0

**Published:** 2021-05-26

**Authors:** C. Homer-Bouthiette, L. Xiao, Marja M. Hurley

**Affiliations:** 1 Yale Internal Medicine Residency Program, Yale New Haven Hospital, New Haven, CT 06510 USA; 2 Department of Medicine, School of Medicine, UConn Health, University of Connecticut, 263 Farmington Ave., Farmington, CT 06030 USA

**Keywords:** Cell biology, Genetics

## Abstract

Fibroblast growth factor 2 (FGF2) is important in musculoskeletal homeostasis, therefore the impact of reduction or *Fgf2* knockout on skeletal muscle function and phenotype was determined. Gait analysis as well as muscle strength testing in young and old WT and *Fgf2KO* demonstrated age-related gait disturbances and reduction in muscle strength that were exacerbated in the *KO* condition. *Fgf2* mRNA and protein were significantly decreased in skeletal muscle of old WT compared with young WT. Muscle fiber cross-sectional area was significantly reduced with increased fibrosis and inflammatory infiltrates in old WT and *Fgf2KO* vs. young WT. Inflammatory cells were further significantly increased in old *Fgf2KO compared with old WT*. Lipid-related genes and intramuscular fat was increased in old WT and old *Fgf2KO* with a further increase in fibro-adipocytes in old *Fgf2KO* compared with old WT. Impaired FGF signaling including Increased *β-Klotho*, *Fgf21* mRNA, FGF21 protein, phosphorylated FGF receptors 1 and 3, was observed in old WT and old *Fgf2KO. MAPK/ ERK1/2* was significantly increased in young and old *Fgf2KO*. We conclude that *Fgf2KO*, age-related decreased FGF2 in WT mice, and increased FGF21 in the setting of impaired *Fgf2* expression likely contribute to impaired skeletal muscle function and sarcopenia in mice.

## Introduction

Age related progressive degenerative loss of skeletal muscle mass affects both its quality and strength^1–4^. An ASBMR topical meeting concluded that these deficits should also lead to a loss of mobility beyond a certain threshold, which increases the risk of further development of sarcopenia^5^. These symptoms are particularly exacerbated in frail elderly patients where sarcopenia becomes rapidly accelerated for causes still unknown. Sarcopenia and age-related loss of bone mass are estimated to cost the health care system billions of dollars annually^6^. Several growth factors and cytokines are associated with the onset and progression of skeletal muscle aging including loss of insulin like growth factor-1 (IGF-1), increases in pro-inflammatory transforming growth factor beta (TGF-β), tumor necrosis factor alpha (TNF-α), and changes in interleukin-6 (IL-6)^7^. FGF2 plays a critical role in adult regenerative myogenesis^8^ however the role of FGF2 in sarcopenia with age is undefined. In support of a principal role for FGF2 in muscle function, neutralizing antibody to FGF2 was shown to attenuate skeletal muscle repair^8^ and intramuscular injection with recombinant FGF2 was reported to improve recovery in dystrophic muscle^9^. As previously noted, FGF2 is important in myogenesis and along with IGF-1, levels were increased in forelimb muscle homogenates from young (8-week-old) mice^10^, however neither IGF-1 or FGF2 were measured in muscle of old mice in this study. Since the impact of Fgf2 germ-line knockout on age-related muscle function or skeletal muscle phenotype has not been reported, we examined whether aging is associated with changes *in Fgf2* expression in skeletal muscle, and whether disruption of the Fgf2 gene in mice results in exacerbation of impaired exercise tolerance and muscle strength and sarcopenia in skeletal muscle with age.

In addition to FGF2, other FGFs are present in skeletal muscle^11–14^ and are active in muscle homeostasis^11^. It has been proposed that perhaps muscle is a determinant of bone loss with aging and that paracrine signaling via local growth factors including FGF2 between muscle and bone could be important^10^. This concept was of interest since disruption of the Fgf2 gene in mice resulted in accelerated age-related bone loss manifested by decreased bone mineral density and bone mineral content, reduced bone formation^15^ and increased bone marrow fat accumulation^16^. Among the other FGF ligands expressed in muscle, the myokine FGF21 is expressed at low levels during muscle homeostasis but is significantly increased in response to exercise as well as pathophysiologic states of mitochondrial stress and aging via binding to FGF membrane bound receptor beta-klotho (*β-Klotho*) as well as tyrosine kinase FGF receptors to activate multiple downstream signaling pathways^14^. Although there has been no functional/biologic association between FGF2 and FGF21, we were intrigued about its expression in skeletal muscle in *Fgf2KO* mice since overexpression of Fgf21 in mice has been associated with phenotypic changes^14^ similar to those observed in aged WT and aged *Fgf2KO* skeletal muscle in the present study. Specifically, chronic FGF21 expression during stress or other myopathies resulted in increased fat accumulation in skeletal muscle^14^ and modulation of adipocyte signaling including increased PPARg protein^17^. Since we previously reported that there was fat accumulation in the bone marrow of *Fgf2KO* mice with age^16^, we examined whether there was accumulation of fat and other markers of sarcopenia in skeletal muscle of *Fgf2KO* and whether there was associated modulation of the myokine FGF21 that has been reported to be associated with increased skeletal muscle fat accumulation and altered adipogenic and FGF receptor signaling.

## Results

### Gait dynamics in young and aged WT and *Fgf2KO* mice

**Figure 1:**
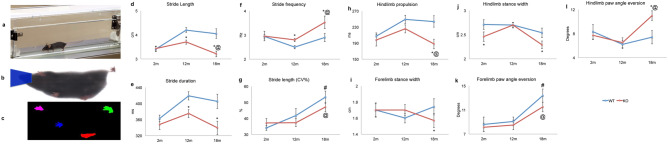
DigiGait analysis of young, middle-aged and old WT and *Fgf2KO* mice. To assess whether knockout of the Fgf2 gene resulted in alteration in gait, treadmill exhaustion test ventral plane videography (DigiGait, Mouse Specifics, Inc., Quincy, MA) was performed. (**a**–**c**) DigiGait related images. (**a**) Lateral view of the mouse containment area around the treadmill belt. (**b**) Representative image of mouse from the camera position below the transparent treadmill belt. The blue area is added during video analysis to nullify any background generated by the nose. (**c**) Representative image from the DigiGait software displaying the remaining pixels representing paw contact with the belt utilized for gait analysis. Gait analysis of young (2 months), middle-aged (12 months) and old (19 months) WT and *Fgf2KO* mice showed: (**d**) Stride length was significantly shorter in old *Fgf2KO* compared to old WT and middle-aged *Fgf2KO.* Middle-aged *Fgf2KO* was also significantly shorter than middle-aged WT. (**e**) Stride duration was significantly shorter in middle-aged *Fgf2KO* compared to middle-aged WT as well as in old *Fgf2KO* compared to old WT. (**f**) Stride frequency significantly was higher in old *Fgf2KO* compared to old WT and middle-aged *Fgf2KO*. Middle-aged *Fgf2KO* also showed significantly increased stride frequency compared to WT of the same age. (**g**) Stride variability (coefficient of variation) was higher in old WT compared to middle-aged WT as well as old *Fgf2KO* compared with middle-aged *Fgf2*KO. (**h**) Hindlimb propulsion was significantly shorter in old *Fgf2KO* versus old WT and middle-aged *Fgf2KO*. (**i**) Forelimb stance width was significantly narrower in old Fgf2*KO* versus old WT. (**j**) Hindlimb stance width was significantly narrower in old *Fgf2KO* versus old WT. (**k**) Forelimb paw angle eversion was similar in young and middle-aged WT and *Fgf2KO* mice and was increased in aged vs. middle-aged in both WT and *Fgf2KO* mice. (**l**) Hind paw eversion (an indicator of muscle weakness) was similar in young and middle-aged WT and *Fgf2KO* mice, but significantly greater in old *Fgf2KO* mice compared with old WT and middle-aged Fgf2KO. n = 6–10 mice per group, *Fgf2KO significantly different from WT of same age, ^#^19 months WT significantly different from 12 months WT, ^@^19 months *Fgf2KO* significantly different from 12 months *Fgf2KO*.

Prior to exercise, body weight (g) of 2-month, 12-month, and 19-month-old WT and *Fgf2KO* mice was measured. There were no significant differences in body weight between young WT and young *Fgf2KO*, between middle-aged WT and *Fgf2KO*, or between old WT and old *Fgf2KO* mice. The DigiGait treadmill apparatus can be seen in Fig. 1a from a lateral view. Representative images from the treadmill capturing camera (Fig. 1b) and the digital paw gait analysis from the DigiGait software (Fig. 1c) is also displayed.

We determined whether there were significant differences in any of the gait parameters with age and or genotype. Stride length (Fig. 1d) and stride duration (Fig. 1e) were similar between young WT and young *Fgf2KO.* There were no significant changes between the young and middle-aged mice, however stride length and stride duration were significantly shorter (p < 0.05) in the middle-aged *Fgf2KO* mice compared to the middle-aged WT mice. Stride length was also significantly decreased in the old *Fgf2KO* mice when compared to the middle-aged *Fgf2KO* group as well as the old WT mice. Stride duration was similar between WT groups of different ages. *Fgf2KO* mice of different age groups showed statistically similar stride duration. When *Fgf2KO* mice were compared to the WT mice, stride duration was significantly shorter (p < 0.05) in both the middle-aged and old age groups. Stride frequency (Fig. 1f) was not significantly different between young WT and young *Fgf2KO,* or between old WT, middle-aged WT and young WT. However, stride frequency was significantly increased in old *Fgf2KO* compared with middle-aged *Fgf2KO*. Significant increases (p < 0.05) in stride frequency were also seen when old *Fgf2KO* were compared with old WT as well as in middle-aged *Fgf2KO* versus middle-aged WT. As shown in Fig. 1g, stride-to-stride variability for stride length CV was not significantly different between young WT and young *Fgf2KO*, middle-aged WT and middle-aged *Fgf2KO*, or between aged WT and old *Fgf2KO*. However, this parameter was significantly different in middle-aged WT compared with old WT as well as middle-aged *Fgf2KO* compared with old *Fgf2KO*. Of particular significance, gait analysis revealed that hindlimb propulsion duration (Fig. 1h) (an indicator of muscle strength) was similar in young WT and *Fgf2KO* mice as well as middle-aged WT and *Fgf2KO* although some decrease was evident. Significantly shorter propulsion duration was observed in old *Fgf2KO* mice (p < 0.05) compared with old *WT* littermates as well as aged *Fgf2KO* group compared to middle-aged *Fgf2KO*. We also examined stance width in forelimbs and hindlimbs, in addition to hind paw eversion. As shown in (Fig. 1i,j), comparison of forelimbs and hindlimbs stance width in young WT and old WT mice revealed no significant age-related changes. Furthermore, no changes were evident between young WT and young *Fgf2KO* or middle-aged WT versus middle-aged *Fgf2KO.* However, when compared to old WT, forelimb and hindlimb stance width was significantly narrower in old *Fgf2KO*, thus suggesting exacerbated gait disturbances specific to the *KO* condition versus normal age-related changes typical of WT animals (p < 0.05). Forelimb paw angle variability (Fig. 1k) was similar in young and middle-aged WT and *Fgf2KO* mice, but was increased in both aged WT and aged *Fgf2KO* mice when compared to their middle-aged counterparts. Similarly, as shown in Fig. 1l, hind paw eversion (paw angle, an indicator of muscle weakness) was similar in young WT and *Fgf2KO* mice, as well as middle-aged WT and *Fgf2KO* mice. Significantly increased hind paw eversion was evident in old *Fgf2KO* mice compared with old WT and middle-aged *Fgf2KO* (p < 0.05). Braking duration (an indicator of ground reaction forces) was similar between young, middle-aged, and old WT and *Fgf2KO* mice (data not shown).

### Forelimb grip strength test in young and adult WT and *Fgf2KO* mice

**Figure 2:**
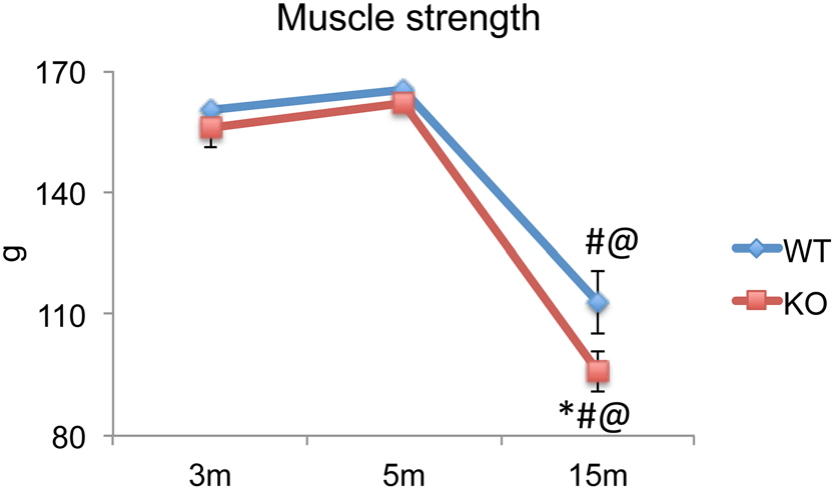
Forelimb grip test during the aging process in female *Fgf2KO* mice. The same mice were subjected to the grip strength test at 3, 5 and 15 months of age. Measurements were performed 3 times per day for 3 days, and the data were averaged as an individual value. Time course of muscle grip force analyzed by the Two-way repeated measure ANOVA, along with Tukey’s multiple comparison test showed that no grip strength differences were observed between 3 and 5 m WT mice, however there was a significant reduction between 3 m WT and 15 m WT as well as 5 m WT and 15 m WT. There were no significant differences between 3 and 5 m WT and 3 m and 5 m *Fgf2KO* mice. There were no significant differences between 3 and 5 m *Fgf2KO* mice, however there was a significant reduction between 3 m *Fgf2KO* and 15 m *Fgf2KO* as well as 5 m *Fgf2KO* and 15 m *Fgf2KO.* There was a further significant reduction of grip strength between 15 m WT and 15 m KO. n = 3 mice/group. *Compared to corresponding WT p < 0.05, ^#^compared to corresponding 5 m p < 0.05, ^@^compared to corresponding 3 m p < 0.05.

We assessed whether other parameters of muscle function such as grip strength was affected by *Fgf2* ablation or with age using the same cohort of WT and *Fgf2KO* mice (Fig. 2). Two-way analysis of variance of grip strength based on age revealed no differences between 3 and 5 m WT mice. However, there was a significant reduction between 3 m WT and 15 m WT (p < 0.000) as well as 5 m WT and 15 m WT (p < 0.000). There were no significant differences between 3 and 5 m WT and 3 m and 5 m *Fgf2KO* mice. There were also no significant differences between 3 and 5 m *Fgf2KO* mice, however there was a significant reduction between 3 m *Fgf2KO* and 15 m *Fgf2KO* (p < 0.000) as well as 5 m Fgf2KO and 15 m Fgf2KO (p < 0.000). There was a further significant reduction of grip strength between 15 m WT and 15 m *Fgf2KO* (p < 0.015).

### Age-related changes in *Fgf2* mRNA and protein expression in skeletal muscle of WT mice

**Figure 3:**
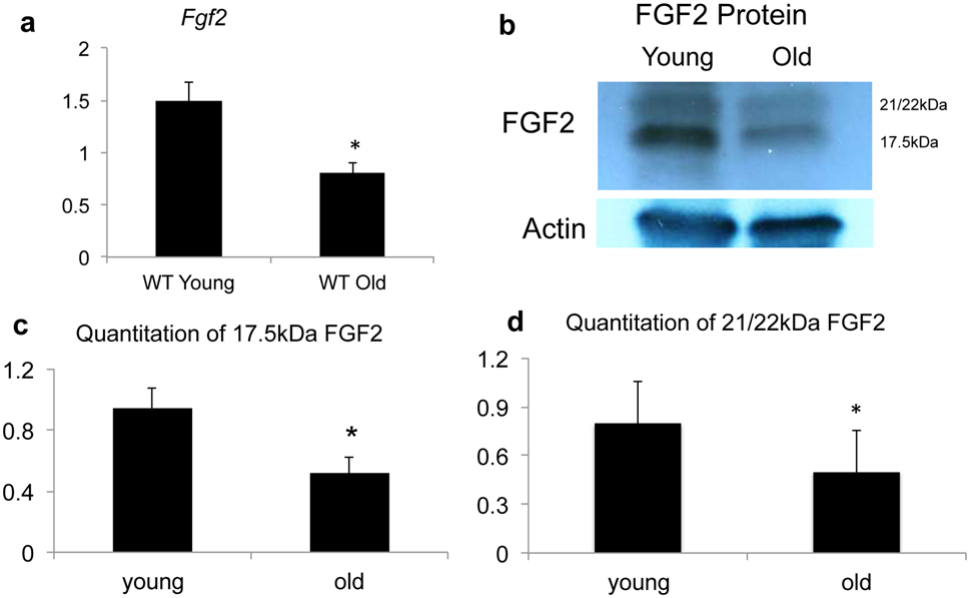
FGF2 expression in skeletal muscle of young and old WT muscle. Young (2 months) and old (18 months) WT mouse hindlimb muscles were isolated and qPCR and Western blotting for *Fgf2* mRNA and FGF2 protein were performed on whole muscle isolates. (**a**) *Fgf2* mRNA relative to actin. n = 6–10 mice/group. (**b**) Representative Western blots for FGF2 protein and (**c**) quantitative analysis of 17.5 kDa FGF2 isoform. (**d**) Quantitative analysis of 21/22 kDa FGF2 isoforms. Quantifications were summed from 3 independent experiments. *Significantly different from young.

Since we observed impaired muscle function in old WT muscle, we examined *Fgf2* mRNA in young versus old WT mice (Fig. 3a). There was a significant decrease in *Fgf2* mRNA in old WT mice compared with young WT (p < 0.05). We also examined changes in FGF2 protein in young and old WT mice. As shown in a representative experiment (Fig. 3b), FGF2 protein isoform bands of 17.5, 21/22 kDa were expressed in muscle and there was a marked decrease in all FGF2 isoforms with age. Quantitative analysis of the 17.5 kDa (Fig. 3c) and 21/22 kDa FGF2 isoforms (Fig. 3d) were determined by Western blot, in 3 independent experiments, and were significantly decreased in the old muscle (p < 0.05).

### Comparison of changes in muscle fiber area, fibrotic tissue and inflammatory cells in WT and *Fgf2*KO mice

**Figure 4:**
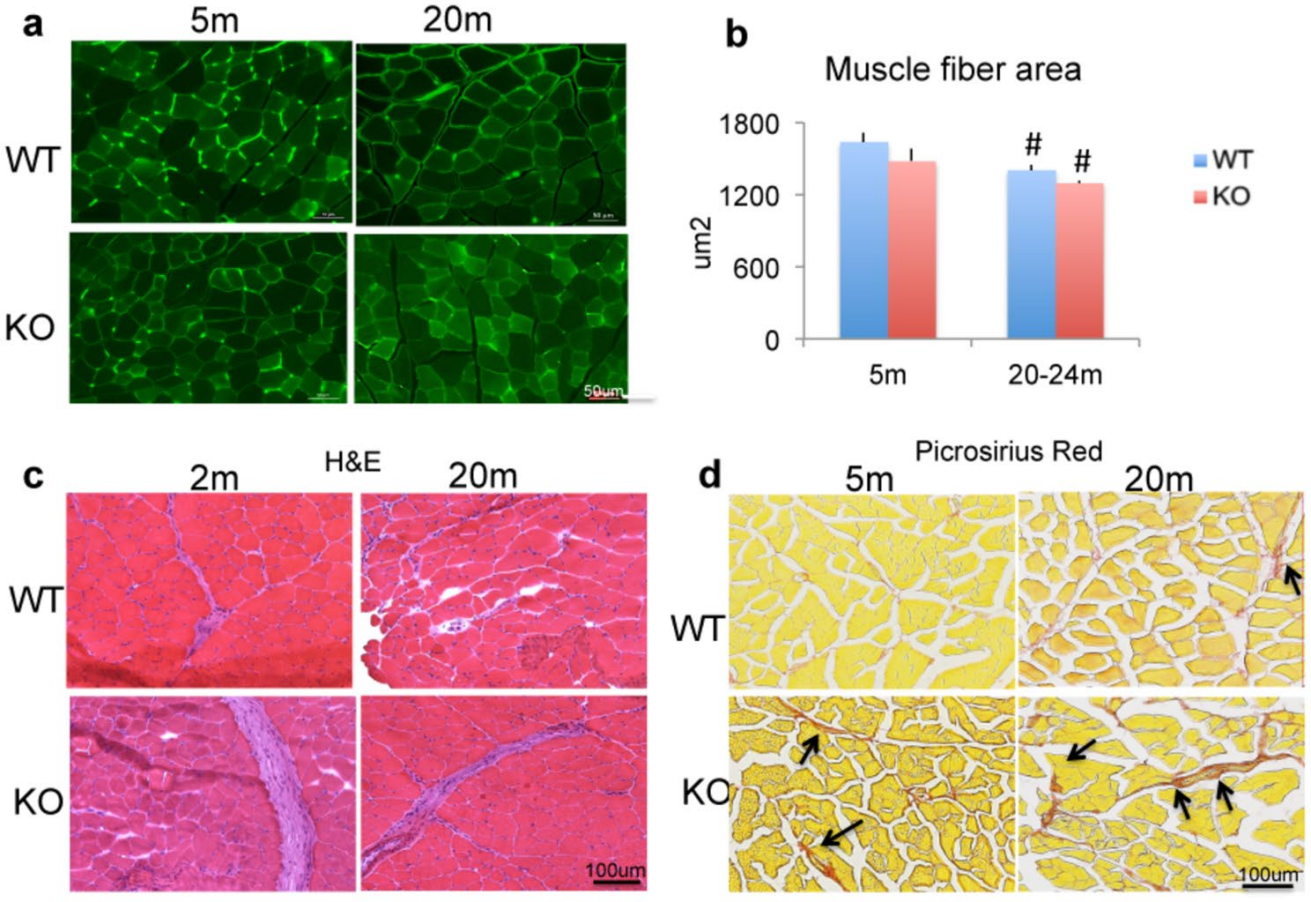
Histomorphometric analysis of muscle from WT and *Fgf2KO* mice. Muscle sections from tibialis anterior muscle in 5-month and 20–24 months old WT and *Fgf2KO* mice. (**a**) Laminin-stained sections reveal overall reduced myofiber cross sectional area is observed within old WT and old *Fgf2KO* mice. (**b**) Quantitative analysis of cross-sectional area of skeletal muscle from 3 mice/group revealed significant reduction in old WT and old *Fgf2KO* compared with respective young genotype. ^#^Compared to corresponding 5 m p < 0.05. (**c**) Muscle sections from tibialis anterior muscle in young and old (2 months and 22 months) WT and *Fgf2KO* stained with hematoxylin and eosin reveal large swaths of ECM/collagen deposition and increased inflammatory infiltrate in young and old *Fgf2KO* as well as old WT. (**d**) Picroriues red staining for collagen showed increased fibrosis (arrows) in the muscle of 20 m-WT and 5 m *Fgf2KO* compared with 5 m WT, and further increased in 20 m *Fgf2KO.*

We assessed whether impaired muscle function in old WT and old *Fgf2KO* was associated with alteration in skeletal muscle fiber area using laminin labeled sections (Fig. 4a). There were no consistent differences between young WT and young *Fgf2KO*. In contrast in both old WT and old *Fgf2KO*, the muscle fiber cross sectional area appeared to be decreased when compared with young muscle from both genotypes. Quantitative measurement of cross- sectional area (Fig. 4b) revealed no significant differences between young WT and young *Fgf2KO,* in contrast fiber cross sectional area was significantly reduced in old WT and old *Fgf2KO* compared with respective young of each genotype.

Based on the gait and strength abnormalities in *Fgf2KO* mice and since sarcopenic phenotype includes fibrotic and inflammatory changes in skeletal muscle**,** we examined whether *Fgf2KO* skeletal muscle exhibited changes observed in sarcopenic muscle. Tibialis anterior muscles were harvested from 2 and 22-month-old WT and *Fgf2KO* littermate mice and stained for H&E. As shown in Fig. 4c, H&E-stained sections of tibialis anterior muscle revealed swaths of extracellular matrix collagen and areas of inflammation in 2-month-old *Fgf2KO* as well as 20-month-old of both genotypes. To confirm fibrosis, sections were stained for collagen using Picrosirius Red and as shown in Fig. 4d there is marked increased collagen in 20 m *WT* and in 5 m *Fgf2KO* compared to 5 m WT, and further increase in 20 m *Fgf2KO.*

**Figure 5:**
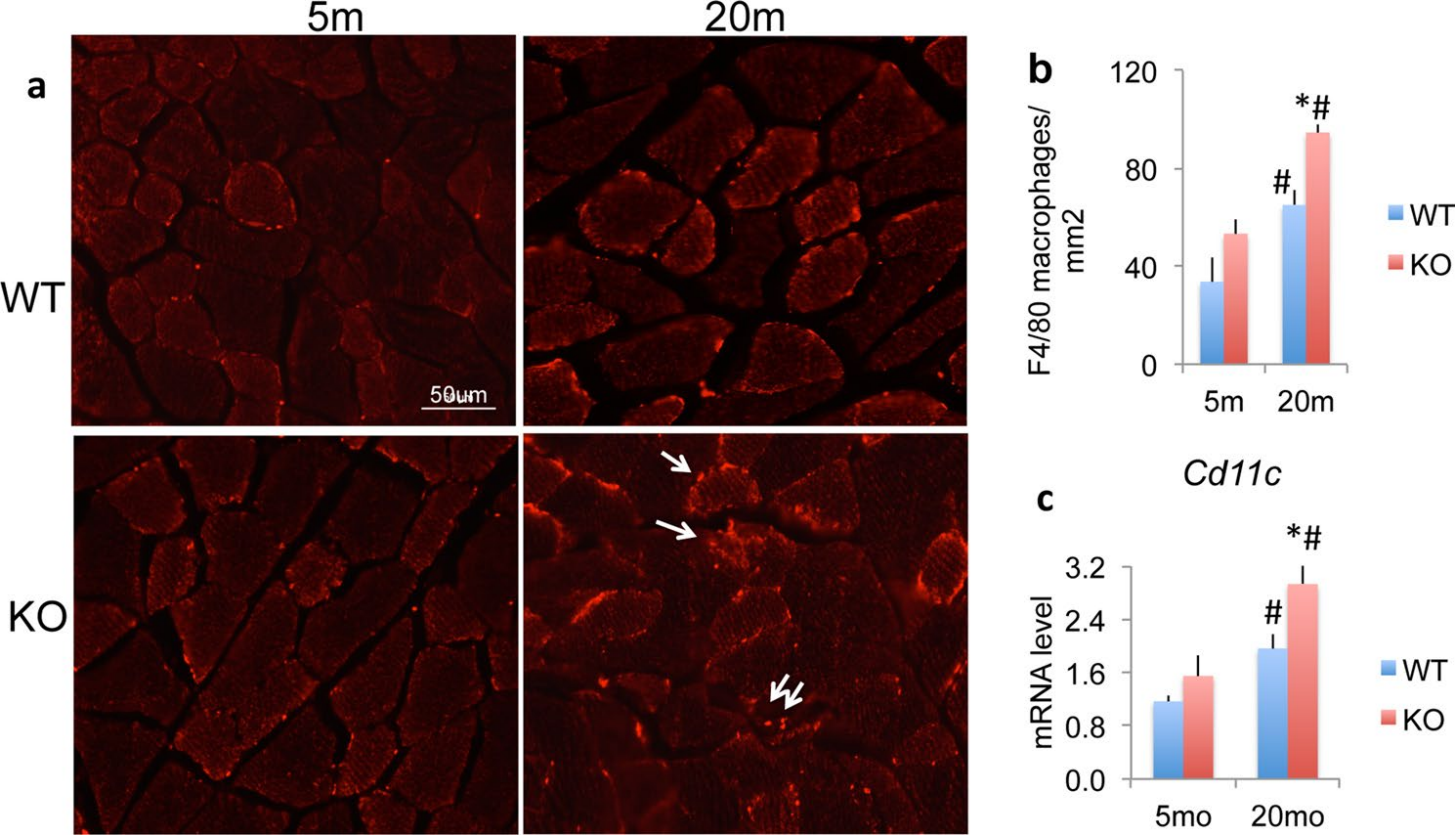
Expression of inflammatory cells in skeletal muscles of *WT* and *Fgf2KO* mice. Muscle sections from tibialis anterior muscle in 5 m and 20 m old WT and *Fgf2KO* mice were used. (**a**) F4/80^+^ macrophages (arrows) in the muscle of mice. Compared with young of both genotypes, there were increased F4/80^+^ cells in old muscle of both genotypes with a further increase in 20 m *Fgf2KO.* (**b**) Quantification of macrophages in the muscle showed reveals a significant increase in macrophages in 20 m WT and 20 m *Fgf2KO* and a further significant increase in 20 m *Fgf2KO* compared with 20 m WT. n = 10 fields/mouse, n = 3 mice/group. (**c**) Cd11c mRNA levels in muscle. n = 4–8 mice/group. *Compared to corresponding WT p < 0.05, ^#^compared to corresponding 5 m p < 0.05.

To confirm inflammatory cells, we performed F4/80 staining of skeletal muscle for macrophages from 5 and 20 m WT and *Fgf2KO.* As shown in Fig. 5a compared with young of both genotypes, there was increased F4/80 labelled cells in old muscle of both genotypes with a further increase in 20 m *Fgf2KO.* Quantitation shown in Fig. 5b reveals a significant increase in macrophages in 20 m WT and 20 m *Fgf2KO* and a further significant increase in 20 m *Fgf2KO* compared with 20 m WT. To determine whether M1 inflammatory macrophages are infiltrating the old *Fgf2KO* skeletal muscle, we performed qPCR analysis on young and old WT and *Fgf2KO* skeletal muscle. As shown in Fig. 5c, there is a significant increase in *Cd11c* mRNA (a M1 macrophage marker gene) in 20 m WT and 20 m *Fgf2KO* and a further significant increase in 20 m *Fgf2KO* compared with 20 m WT.

### Comparison of adipocyte related genes and intramuscular fat accumulation in *WT* and *Fgf2KO* mice

**Figure 6:**
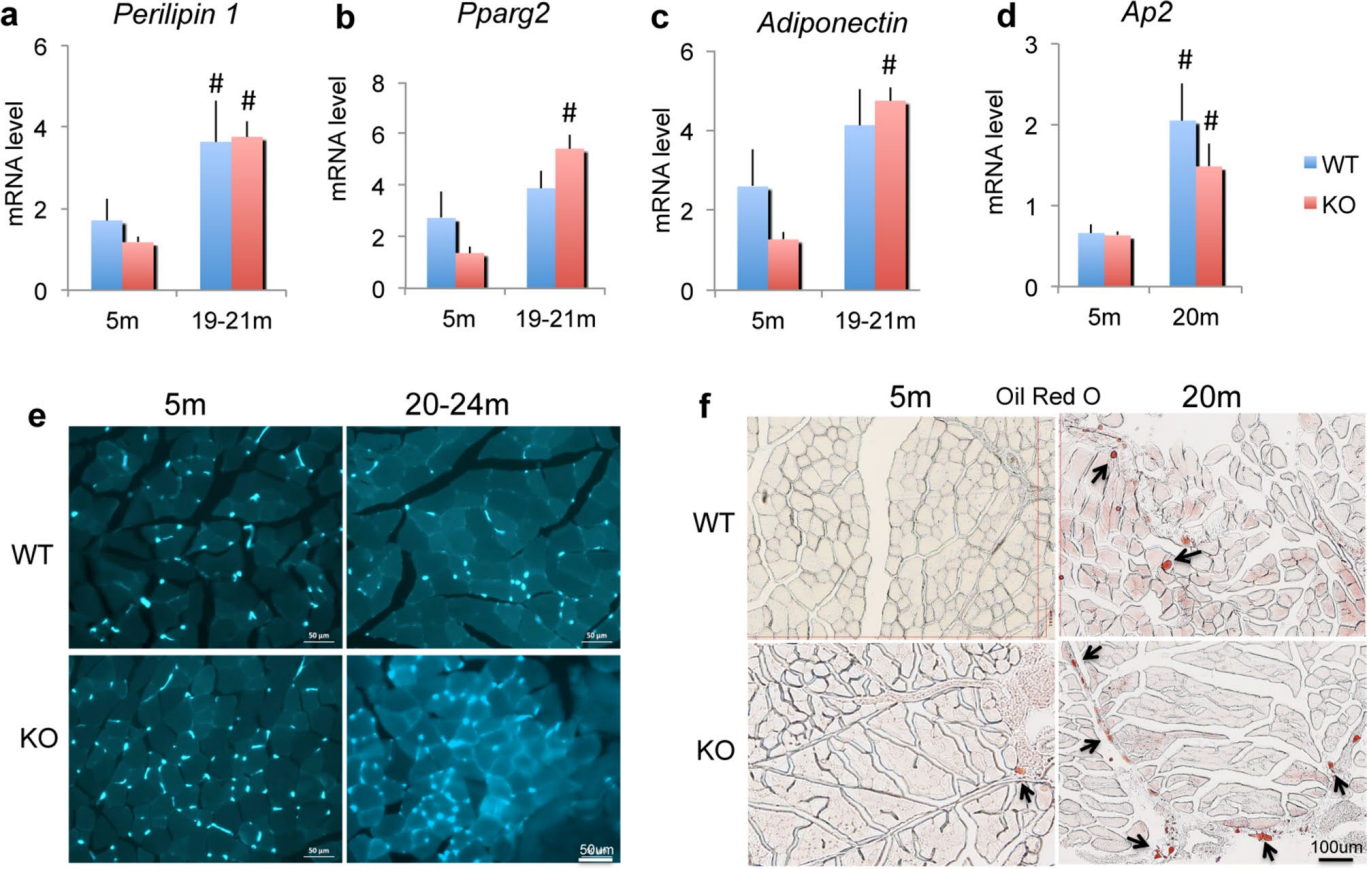
Expression of mRNA for adipocyte related genes and intramuscular fat accumulation in WT and *Fgf2KO* mice. Young (5 m) and old (20–24 m) WT and Fgf2*KO* mouse hindlimb muscles were isolated and total RNA extracted from whole muscle for qPCR. (**a**) *Plin1* mRNA was significantly increased in old WT and old *Fgf2KO* relative to young of both genotypes. (**b**) *Pparg2* mRNA, (**c**) *Adiponectin* mRNA was significantly increased in old *Fgf2KO* relative to young *Fgf2KO* (**d**) *Ap2* mRNA was significantly increased in old WT and old *Fgf2KO* compared with young of both genotypes*.* n = 4–8 mice/group. ^#^Compared to corresponding 5 m p < 0.05. (**e**) AP2/GFP labeling is observed in young and old muscle of both genotypes but markedly increased staining is observed in muscle from old *Fgf2KO* mice compared with old WT. (**f**) Oil Red O staining showed markedly increased intramuscular fat tissue (arrows) in 20 m WT and 20 m *Fgf2KO* compared to 5 m-*WT.*

We previously reported increased fat accumulation in the bone marrow of *Fgf2KO* mice with age^16^, we therefore examined whether there were changes in the expression of lipid related genes. Specifically, the mRNAs for the lipid droplet proteins perilipin 1 through 5 (Plin), Pparg2, Adiponectin and Ap2, as well as fat accumulation with age in muscle of WT and *Fgf2KO* mice was determined. As shown in Fig. 6a, there was significant increase in *Plin1* mRNA in gastrocnemius muscle from old WT and *Fgf2KO* mice. There were no significant differences in expression of *Plin-2*, *Plin-*3, *Plin-*4 or *Plin-*5 mRNA (data not shown). As shown in Fig. 6b,c respectively, relative to young WT, *Pparg2* and *Adiponectin* mRNA were increased in old WT but were not significant. In contrast, *Pparg2* and *Adiponectin* mRNA were significantly increased in old *Fgf2KO* compared with young *Fgf2KO***.** *Significantly increased* Ap2 mRNA (Fig. 6d) was observed skeletal muscle of *old WT and old Fgf2KO compared with young of both genotypes. Increased* AP2/GFP staining was observed in inter-fiber cells of muscle of both old WT and *Fgf2KO* (Fig. 6e) but no major differences in AP2 labeling was observed in young WT versus young *Fgf2KO.* However, a striking increase in AP2 labeling was observed in old *Fgf2KO* compared with old WT. Oil Red O staining (Fig. 6f) showed markedly increased intramuscular fat droplets in muscle of 20 m WT and 20 m *Fgf2KO.*

### Age and genotype-related changes in β-Klotho receptor, Fgf21 expression in skeletal muscle of WT and* Fgf2KO *mice

**Figure 7:**
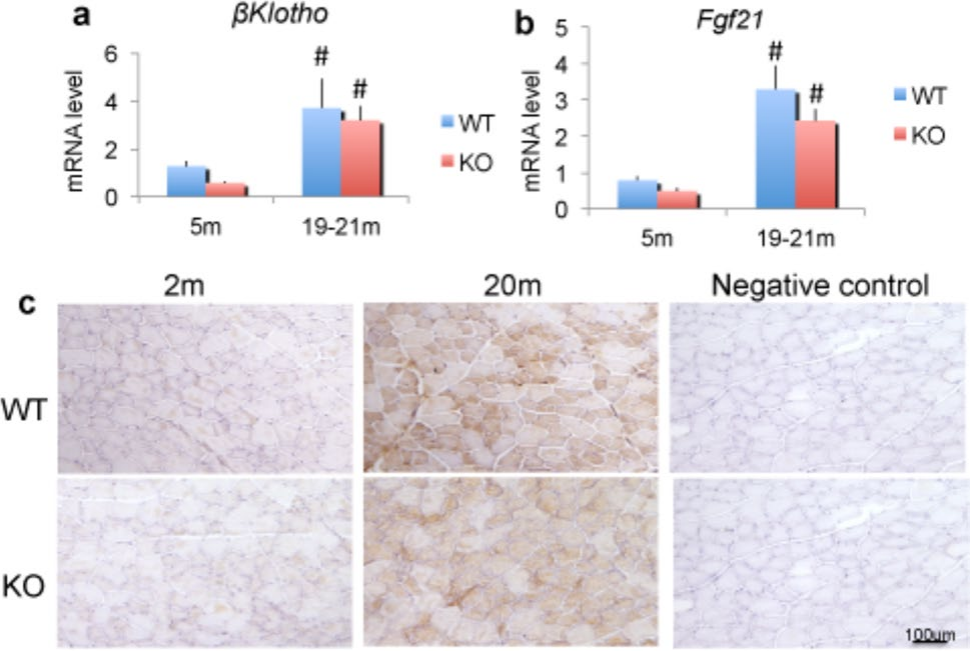
Expression of *β-Klotho* mRNA, *Fgf21* mRNA and FGF21 protein in skeletal muscle from WT and *Fgf2KO* mice. Young (5 m) and old (20 m) WT and *Fgf2KO* mouse hindlimb gastrocnemius muscles were isolated and total RNA extracted for qPCR (**a**) *β-Klotho* mRNA; (**b**) *Fgf21* mRNA were significantly increased in old muscle of both genotypes n = 4–6 mice/group. ^#^Compared to corresponding 5 m p < 0.05 (**c**) Young (5 m) and old (20 m) WT and *Fgf2KO* mouse tibialis anterior muscles were isolated, sectioned and immunohistochemistry for FGF21 revealed increased FGF21 in muscle cells of old WT and *Fgf2KO* compared with young of both genotypes.

We assessed whether reduction of FGF2 in old WT or knockout of FGF2 differentially modulated the pro-adipogenic growth factor FGF21 and its receptor β**-**Klotho. *β****-****Klotho* mRNA (Fig. 7a) was significantly increased in both old WT and old *Fgf2KO* compared with young WT and young *Fgf2KO. Fgf21* mRNA was significantly increased in both old WT and old *Fgf2KO* compared with young of both genotypes. (Fig. 7b). Immunohistochemistry revealed that FGF21 protein was also markedly increased in both WT and old *Fgf2KO* muscle compared with young of both genotypes (Fig. 7c). However, comparing the *Fgf21* mRNA expression levels in the skeletal muscle of young WT versus the old WT (0.79 vs. 3.39) represents a fourfold increase in *Fgf21* mRNA in the old WT. In contrast, comparing Fgf21 mRNA in the young *Fgf2KO *vs.* old Fgf2KO *(0.49 vs. 2.43) represents approximately fivefold increase in the old *Fgf2KO.* However, there is no significant difference in *Fgf21* mRNA expression in old WT versus old *Fgf2KO.* Similarly, there was approximately threefold increase in *β-Klotho* mRNA in old WT compared with young (3.70 vs. 1.28), but approximately sixfold increase in *β-Klotho* mRNA in old *Fgf2KO* compared with young (3.20 vs. 0.57). There was no significant difference in *β-Klotho* mRNA of old WT versus old *Fgf2KO.*

### Age and genotype-related changes in activated FGF receptors and MAPK/ERK kinase signaling in skeletal muscle of WT and* Fgf2KO *mice

**Figure 8:**
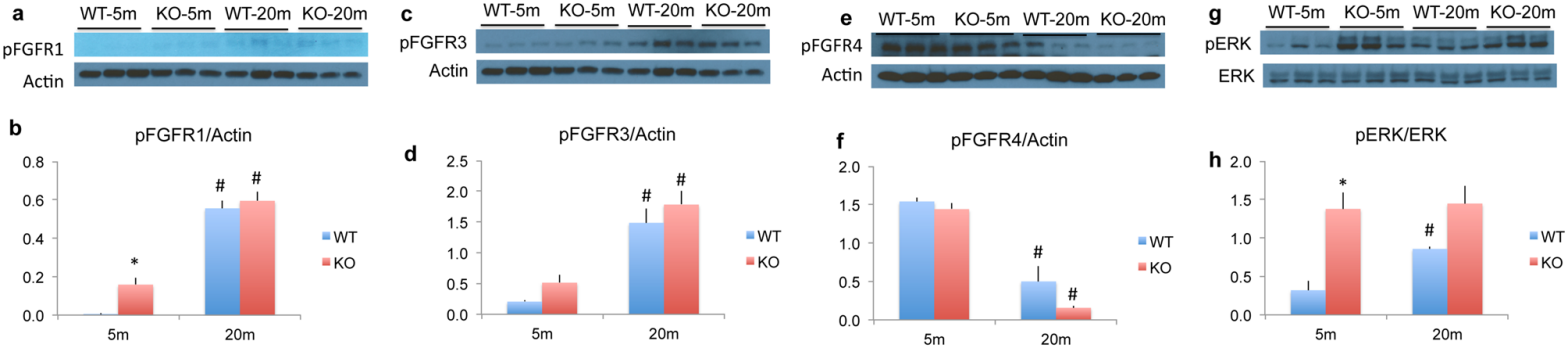
Expression of *FGFR* protein and MAPK/ERK signaling in skeletal muscle from WT and *Fgf2KO* mice. Young (5 m) and old (20 m) WT and *Fgf2KO* mouse triceps muscles were isolated and total protein extracted for western blotting. (**a**) phosphoFGFR1 Western blot and (**b**) quantification. (**c**) phosphoFGFR3 Western blot and (**d**) quantification. (**e**) phosphoFGFR4 Western blot and (**f**) quantification. (**g**) phosphoERK and total ERK Western blot and (**h**) quantification**. **(**a**,**b**) phosphoFGFR1; (**c**,**d**) *phosphoFGFR3* were significantly increased, while (**e**,**f**) phosphoFGFR4 was significantly decreased in old muscle of both genotypes. (**g**,**h**) phosphoERK1/2 was significantly increased in 20 m old as well 5 m and 20 m *Fgf2KO* compared with 5 m WT. n = 3 mice/group. *Compared to corresponding WT p < 0.05, ^#^compared to corresponding 5 m p < 0.05.

We also determined by western blot whether there were changes in activated/phosphorylated FGF receptors and downstream signaling molecules phosphorylated extracellular signal regulated kinases (ERK1/2) and protein kinase B (AKT). As shown in Fig. 8 we observed significantly increased phosphoFGR1 (Fig. 8a,b) and phosphoFGFR3 (Fig. 8c,d) but significantly decreased phosphoFGFR4 (Fig. 8e,f) in 20 m WT and 20 m *Fgf2KO* compared with 5 m of both genotypes. MAPK phosphoERK1/2 was significantly increased in 20 m WT compared with 5 m WT and was also significantly increased in 5 m and 20 m *Fgf2KO *compared with 5 m WT (Fig. 8g,h). PhosphoAKT was not significantly altered in old mice of either genotype (data not shown).

## Discussion

FGF2 is important in bone^21^ as well as muscle homeostasis and function and since muscle function as determined by gait analysis has not been previously reported in *Fgf2KO* mice, we assessed the importance of reduced or lack of skeletal muscle FGF2 on gait performance in young and middle-aged versus old mice of both genotypes. The data presented herein represents the first quantitative analysis of gait in *Fgf2KO* mice. It is interesting to note that we did not observe gait disturbances in either young or aged *Fgf2KO* mice in their cages, thus the treadmill gait analysis was very enlightening. The middle-aged mice showed increases in gait performance over the young mice of the same genotype across all gait metrics due to increased musculature as they reach full adulthood, which was also observed by Wooley et al.^22^. The ~ 15% shorter stride length deficit between old *Fgf2KO* and old WT and further ~ 13% decrease in old *Fgf2KO* versus middle-aged *Fgf2KO* are considered to be very robust deficits. A ~ 4% deficit in stride length was observed in the old WT when compared to the middle-aged WT indicating the presence of FGF2 can maintain healthy muscle strength relative to the *Fgf2KO.* As previously reported, in an otherwise healthy patient with neuromuscular disease, a ~ 10% reduction in stride length could be clinically relevant^23^.

In order to compare across age and genotype, the treadmill speed was set to 10 cm/s for young, middle-aged, and old WT, as well as for *Fgf2KO* mice. The shorter stride length observed with age and genotype is consistent with a significant increase in stepping frequency by the old *Fgf2KO* mice. Previous studies showed that increased stepping frequency as part of daily living results in increased cardiopulmonary activity^23^. We observed a significant narrowing of forelimb and hindlimb stance width in old *Fgf2KO* compared with old WT. These results suggest that loss of FGF2 affects the forelimb and hindlimb stance, which could contribute to the gait disturbances observed in *Fgf2KO* mice.

Gait analysis revealed that hindlimb propulsion duration (an indicator of muscle strength) was similar in young WT and *Fgf2KO,* but showed a ~ 10% deficit in middle-aged *Fgf2KO* compared to WT of same age, and was significantly shorter in old *Fgf2KO* compared with old WT littermates. The gait disturbance in hindlimb propulsion was exacerbated in the *Fgf2KO* resulting in a ~ 17% decrease from 12 to 19 months compared to a 2% decrease in the WT from the same age range. The aged WT mice are only beginning to show signs of muscle deficits in the fore and hind limbs at 19 months even with the decrease in FGF2 protein in the muscle. This indicates the lower production of FGF2 with age may be at sufficient levels to initiate stem cell mediated repair of muscle fibers and delay the significant age-related effects in the absence of FGF2, observed in the *Fgf2KO*. It is also interesting that hind paw eversion/paw angle (an indicator of muscle weakness of the hind limbs) was similar in young WT compared to young *Fgf2KO* as well as middle-aged WT versus middle-aged *Fgf2KO* mice. However, hind paw eversion was significantly greater in old *Fgf2KO* mice compared with old WT. Thus, in summary, comparison of young WT and young *Fgf2KO* revealed no gait-specific impairment. Some early evidence of gait disturbances could be observed in the middle-aged *Fgf2KO* compared to the WT of the same age. However, several age-related gait changes emerged in old cohorts and were exacerbated in the *Fgf2KO* mice. Age-related gait changes (stride length, stride frequency, stride duration and propulsion, stance width and paw angle) emerged that were exclusive to the *Fgf2KO*. Furthermore, while parameters such as hindlimb propel were shared between young WT and *Fgf2KO*, increased gait defects were evident in aging *Fgf2KO* suggesting that lack of FGF2 further contributes to weakness of hind paws or lower limb muscles.

Similar to the gait analysis in young WT and young *Fgf2KO,* we did not observe any differences in forelimb grip strength in 3 m and 5 m WT and *Fgf2KO*, however, we did observe a significant reduction in forelimb grip strength with age in WT and *Fgf2KO*. Our finding of a further significant reduction in grip strength between 15 m WT and 15 m *Fgf2KO* supports an effect of the *Fgf2KO* in the aged condition.

In the present study, we found that *Fgf2* mRNA and all FGF2 protein isoforms were decreased with age in muscle from WT mice. The single copy of *Fgf2* gene encodes for three protein isoforms in mice, these include a low molecular weight isoform (17.5 kDa) due to translation from AUG codon and two high molecular protein isoforms (21 and 22 kDa) that are translated from CUG codons in-frame with the AUG codon^21^. The low molecular weight isoform is exported from cells and functions in an autocrine/paracrine manner, while the high molecular protein isoforms reside in the nucleus and function in an intracrine manner^21^. In old skeletal muscle, we observed a parallel decrease in all protein isoforms consistent with the observed decrease in message level. This suggests that the decreased isoform levels in skeletal muscle of aging animals is transcriptionally controlled and not due to translational control of the specific isoforms. These observations are novel since there are no reports of decreased FGF2 protein isoform expression in whole skeletal muscle with age. It should be noted that a recent paper reported that in aged single skeletal muscle fibers, *Fgf2* mRNA was increased and that examination of immune-stained FGF2 positive areas in transverse muscle sections demonstrated increased muscle fiber associated FGF2 but decreased interstitial associated FGF2^10^, although Western blots for FGF2 protein were not performed in that study.

Impaired muscle strength was observed in the old WT and old *Fgf2KO* mice, we therefore assessed whether there was evidence of sarcopenic phenotype in skeletal muscle based on age and genotype. We examined muscle fiber size in young and old WT and *Fgf2KO* since previous studies have shown that reduced skeletal muscle function is associated with decreased fiber cross-sectional area^24^. We observed no significant differences in skeletal muscle fiber size in young WT and young *Fgf2KO* in which we had observed no muscle dysfunction. However, muscle dysfunction in old WT and old *Fgf2KO* was associated with significant reduction in fiber size. We also observed changes in other markers of sarcopenia in old WT and old *Fgf2KO* mice including increased fibrosis and inflammatory cells, as well as significantly increased mRNA for the adipogenic-related genes, Perilippin-1, *Ap2 and adiponectin* in old WT and old *Fgf2KO* mice. Interestingly we observed a significant increase in *Pparg2* mRNA in old *Fgf2KO* compared with young *Fgf2KO,* which is consistent with our published studies of increased *Pparg2* mRNA in bone marrow adipocytes in old *Fgf2KO* mice^16^.

As detailed in methods, we crossed the *Fgf2KO* mouse with the aP2/GFP mouse line that contains a 7.6 kb promoter fragment from the adipocyte specific marker gene adipocyte protein 2 (aP2/FABP4) to express the topaz variant of enhanced green fluorescent protein (aP2/Tpz)^19^. The construct is expressed in actively forming brown and white fat in vivo. Therefore, as expected, we observed AP2/GFP expression in young and old mice of both genotypes. However, the fact that we observed a greater increase in AP2-labeling*, Ap2* mRNA in the old WT and old *Fgf2KO* as well as increased Oil Red O staining for fat *s*upports previous studies that demonstrate abnormal adipocyte-like cells accumulation in aged muscle^25,26,27^. The fact that relative to the old WT, there was a further marked increase in fibro-adipocytes in old *Fgf2KO* muscle is interesting and suggest a further exacerbation of the phenotype due to the complete loss of FGF2.

Since we observed no gait dysfunction between young WT and young *Fgf2KO* mice, it is possible that another FGF ligand compensated for the lack of FGF2 in the young *Fgf2KO* mice. In addition to FGF2, previous studies reported that *Fgf5*, *Fgf6* and *Fgf7* are expressed in skeletal muscle^11,28,29^. Consistent with these earlier reports, we observed expression of the mRNA for these FGF ligands in our study (data not shown). FGF6 has been previously reported to have similar effects to FGF2 in muscle homeostasis^30^, however, our results did not demonstrate a compensatory increase in FGF6 in skeletal muscle for FGF2 loss either with age or because of the absence of FGF2 in the knockout condition (data not shown). Interestingly another FGF ligand, *Fgf21* mRNA and FGF21 protein were significantly increased in skeletal muscle of both old WT and old *Fgf2KO.* Previous studies reported the presence of FGF21 in skeletal muscle^31^ and since FGF21, which is typically induced by different kinds of stress, plays various roles in energy metabolism and injury protection as a hepatokine, adipokine, and myokine in an endocrine or autocrine/paracrine manner^14,32^, it is interesting to speculate whether its increase in old *Fgf2KO* and old WT represents a compensatory increase in response to age related muscle dysfunction observed in these mice.

We also observed differential modulation of *β-klotho* in old muscle of WT and *Fgf2KO* compared with young of both genotypes. The mRNA for *β-klotho*^33^, the co-receptor for *Fgf21* was significantly increased in old WT and old *Fgf2KO. Since* we observed a marked increase in FGF21 ligand that signals via *β-klotho,* this suggest that reduced or knockout of *Fgf2* may modulate *Fgf21* and *β-klotho* expression with age. Also, of potential relevance, we observed an increase in skeletal muscle fibro/adipogenic (FAP) cells as shown by increased AP2 staining in old WT and a further increase in old *Fgf2KO.* This observation is noteworthy since previous studies reported that *β-Klotho* transcript expression was strongly upregulated in FAPs entering adipogenic differentiation^33^. Although overexpression of *β-Klotho* in mouse cell line models enhanced adipogenesis in NIH3T3 fibroblasts but had no effect on C2C12 myogenic cells, these studies suggested a pro-adipogenic role for *β-Klotho* in skeletal muscle fibro/adipogenesis and supports further research on involvement of the FGF-FGFR-β-Klotho axis in the fibro/adipogenic infiltration associated with functional deterioration of skeletal muscle in aging^33^.

FGF2 signals via activation of multiple tyrosine kinase FGFR resulting in modulation of several downstream signaling pathways including Ras/MAPK^21^ and all FGFR are expressed in skeletal muscle^12,^^21^. Loss of FGFR1 signaling was previously reported to reduce skeletal muscle mass and disrupts myofiber organization in the developing Limb^34^. In addition since studies have shown that besides *β-Klotho* which Is required for FGF21 signaling^14^, there is preferential activation through FGFR1 and FGFR3, we determined whether there was differential expression/activation of all 4 FGFRs. Consistent with previous reports^14^, we observed increased expression of the active phosphorylated FGFR1 and FGFR3 proteins in old WT and old *Fgf2KO* muscle that may reflect a compensatory effect of the FGF21 signaling pathway in response to the abnormal phenotype observed in skeletal muscle of old WT and *Fgf2KO* mice. Another noteworthy observation was the significant reduction in phospho-FGFR4 in skeletal muscle of old WT and aged *Fgf2KO* since recent studies showed that FGFR4 is required for effective muscle regeneration in vivo^35^. This finding is interesting and could be relevant to the impaired muscle function observed in old WT as well as old *Fgf2KO.* Besides significantly increased phosphoFGFR1 in old *Fgf2KO* muscle as well as old WT, we also observed significantly increased phosphorylated FGFR1 in young muscle from *Fgf2KO* suggesting an effect of both age and genotype modulation of FGFR1.

Alterations in phosphorylated mitogen activated kinases, such as increased pERK1/2 in old muscle compared to young under resting conditions have been reported^7^. Similar to these reports we observed increased pERK1/2 in young and old *Fgf2KO* as well as old WT which could reflect downstream activation by FGFR1. Studies by Moyers et al. demonstrated early signaling events triggered by FGF21 treatment of 3T3-L1 adipocytes including phosphorylation of AKT and ERK1/2 that are also required in the early steps of adipogenesis and increased PPARg^17^ which is the main driver of adipocyte differentiation. In contrast to the studies of Moyers et al.^17^, although we observed increased FGF21, β-Klotho and increased pERK1/2 and PPARg we did not observe increased phospho-FGFR2 or phospho-AKT.

The novel findings in the present study indicate that, in skeletal muscle, FGF2 has a more profound role in muscle repair and aging processes than in early development as the differences in skeletal muscle strength were not observed in the young mice. Our data suggests that there is a natural decline of all FGF2 isoforms in muscle that correspond with inflammatory and fibrotic changes with age that affect muscle function and this process is accelerated in Fgf2*KO* mice. Finally, we conclude that knockout of *Fgf2* exacerbates age-associated changes in muscle phenotype consistent with sarcopenia likely contributing to impaired muscle function in mice.

## Methods

### Animals

The development of the *Fgf2KO* mice on a black-swiss/129 background^18^ as well as detailed characterization of their bone phenotype was previously reported^15,16^. Since we reported that there were increased bone marrow adipocytes with age in *Fgf2KO* mice^16^ and to assess whether there was accumulation of adipogenic marker AP2 in skeletal muscle of *Fgf2KO* mice we utilized mice that are homozygous for the reporter construct *aP2/GFP* and mice that are *Fgf2* null in which both copies of the *Fgf2* gene are deleted. The aP2/GFP mouse line contains a 7.6 kb promoter fragment from the adipocyte specific marker gene adipocyte protein 2 (aP2/FABP4) to express the topaz variant of enhanced green fluorescent protein (aP2/Tpz)^19^. The construct is expressed in actively forming brown and white fat in vivo as well as macrophages in the bone marrow and spleen. Intercrossing of these mice resulted in *Ap2*^*Tg/*+^*/Fgf2*^+*/−*^ mice that are heterozygous at both loci. The heterozygous F1 mice were backcrossed to homozygous AP2^Tg/Tg^ mice. Resulting offsprings that are homozygous for AP2/GFP and heterozygous at the *Fgf2* locus were used to breed to generate the *Ap2* positive WT (Ap2Cyan^Tg/Tg^;Fgf2^+/+^) and *Ap2* positive *Fgf2KO* (Ap2Cyan^Tg/Tg^;Fgf2^−/−^) mice to be used in the experiments. WT and *Fgf2KO* littermates were aged in the transgenic facility at the UCONN Health. Female mice and aged cohorts were used unless stated otherwise.

All animal procedures were approved by the UCONN Health Institutional Animal Care and Use Committee and all experiments were performed in accordance with relevant guidelines and regulations. Additionally the studies complied with the ARRIVE guidelines.

### Muscle performance

To assess whether knockout of the Fgf2 gene resulted in alteration in gait disturbances, treadmill exhaustion test ventral plane videography (DigiGait, Mouse Specifics, Inc., Quincy, MA) analysis was performed whereby young (2-month-old), middle-aged (12-month-old), and old WT (19-month-old) and Fgf2*KO* female mice ran on a motor-driven transparent treadmill belt. Mice were run on 3 consecutive days and results were summed/averaged. Gait dynamics were recorded at 125 frames/second using a high-speed digital video camera (BASLER A310f) and analyzed, as previously described^20^. Briefly, dynamic gait signals consisted of stride, stance and swing duration measures. Stance duration was further subdivided into braking and propulsion duration. Stride frequency was calculated from the number of gait signals over time, and stride length was calculated from the equation: (speed = stride frequency × stride length). Forelimb and hind limb stance width and paw placement angles were additionally calculated^20^. Gait data were collected and pooled from both the left and right forelimbs, and the left and right hind limbs. Measures of stride-to-stride variability (gait variability) for stride length and stance width were determined as the standard deviation and the coefficient of variation (CV). Prior to video capture, mice were allowed to explore the treadmill compartment for several minutes, and received a brief training session with the treadmill in motion for acclimatization to the apparatus. Mice were initially subjected to a range of walking speeds to determine suitable comparative gait parameters. To compare young and old mice, 10 cm/s was identified as the optimal speed for providing consistent analysis between the 2 month and 19-month-old cohorts. Four seconds of video images were selected for each animal, running at a speed of 10 cm/s to provide more than 7 sequential strides.

### Forelimb grip strength testing

A Grip Strength Meter (Bioseb, Pinellas Park, FL) was used to measure maximum muscle strength. Each mouse was allowed to grasp the steel grid connected on the force gauge using its forepaws. The gauge was reset to 0 g after stabilization and the mouse’s tail was slowly pulled back by the operator. Tension was recorded by the gauge at the time the mouse released its forepaws from the grid. The procedure was conducted at a constant speed to allow the mice to build up a resistance against it. Three consecutive measurements per day at 1-min interval were performed in the same cohort of 3-month-old and 5-month-old and 15-month-old WT and *Fgf2KO* mice. To ensure validity of results, each mouse was tested 3 times on 3 consecutive days.

### Histological analysis

Tibialis anterior muscle was examined histologically. Cryosections (10 μm) were cut using an IEC cryostat, collected on Superfrost Plus slides (Fisher Scientific), and subjected to hematoxylin and eosin staining, PicroSirius Red staining for collagen visualization, and oil red O staining for evaluation of intramuscular fat infiltration.

For PicroSirius Red staining, sections were fixed in formalin, then stained in picro-sirius red solution [0.5 g Sirius red F3B (Sigma, St. Louis, MO) dissolved in saturated aqueous solution of picric acid (Sigma, St. Louis, MO)] for 1 h, washed in two changes of acidified water (5 mL acetic acid in 1 L of water), dehydrated in three changes of 100% ethanol, cleared in xylene and mounted in a resinous medium.

For Oil Red O staining, sections were fixed in formalin, then stained in Oil Red O solution for 10 min, washed in two changes of water, stained in Meyer’s Hematoxylin solution, washed in tap water, then in bluing solution for 30 s, washed in tap water, then mounted in 50% glycerol in PBS.

### Immunofluorescence staining

Tibialis anterior muscles were harvested from 5 and 20-month-old WT and Fgf2*KO* mice and frozen sections (10 μm) were washed at room temperature in 1× PBS for 5 min and permeabilized in 0.03% Triton X in PBS for 15 min, then washed in 1× PBS for 5 min, followed by power-blocking for 10 min at room temperature. Samples were incubated with anti-laminin 2 alpha primary antibody (ab11576, Abcam, Cambridge, UK) or anti-F4/80 primary antibody (ab6640, Abcam, Cambridge, UK) in blocking solution (PBS 1% BSA 1% NGS) and incubated at 4 °C overnight, then washed in PBS, 3 × 5 min. The sections were incubated with appropriate fluorescent labeled secondary antibody or 1 h at room temperature then washed in PBS, 3 × 5 min. Samples were mounted with glycerol/PBS and slides were stored at 4 °C. Osteomeasure was utilized to trace fiber area (150 fibers/section) using laminin-stained slides and to count macrophages using F4/80 stained slides.

### Immunohistochemistry for FGF21

Methods utilized were previously published^36,37^. Tibialis anterior muscle were harvested from 5 and 20-month-old WT and Fgf2*KO* mice and frozen sections (10 μm) were used. After sections were washed at room temperature in 1× PBS for 5 min, endogenous peroxidase activity was then blocked by incubating sections with 3% hydrogen peroxide in water for 15 min. Following blocking sections with 10% serum for 1 h at room temperature, the slides were incubated with rabbit anti-Fgf21, 1:250 (ab171941, Abcam, Cambridge, UK) at 4 °C overnight. After washing with TBS containing 0.1% Tween 20, the biotinylated secondary antibody was applied at room temperature for 30 min. Finally, slides were washed and developed with DAB Peroxidase Substrate kit (Vector Laboratories, Burlingame, CA), and counterstained with Harris hematoxylin.

### mRNA isolation and quantitative PCR analysis

Methods utilized were previously published^36,37^. Total RNA was extracted from tibialis anterior and/or gastrocnemius muscle harvested from young (2 months) and old (18 months) WT mice using Trizol reagent. For real-time quantitative reverse transcription PCR analysis, the RNA to cDNA EcoDry Premix Kit (Clontech Inc., Takara Bio, Mountain View, CA) was used to reverse transcribe the RNA to cDNA. iTaq Universal SYBR Green Supermix (Bio-Rad Laboratories, Hercules, CA) and a MyiQ instrument (Bio-Rad Laboratories) were used for quantitative PCR (qPCR). The relative change in mRNA level was normalized to the mRNA level of β-actin, which served as an internal reference for each sample. Fgf2 mRNA level was examined in muscles from young and aged WT mice. The primer sequences for the genes of interest are shown in Table 1.

**Table 1:** Primers used in quantitative real time-PCR.

Gene	Forward	Reverse
*Fgf2*	5′-GTCACGGAAATACTCCAGTTGGT-3′	5′-CCCGTTTTGGATCCGAGTTT-3′
*Fgf21*	5′-CACCGCAGTCCAGAAAGTCT-3′	5′-AATGACCCCTGGCTTCAAGG-3′
*Pparg2*	5′-GCTGTTATGGGTGAAACTCTG-3′	5′-ATAAGGTGGAGATGCAGGTTC-3′
*β-Klotho*	5′-ATAGTTACAACAACGTCTACCGC-3′	5′-CGCCCACGATATGGAGAAGC-3′
*Plin1*	5′-AGGGCCATGTCCCTATCCG-3′	5′-GCGGCACATAGTGTACCACAG-3′
*Adiponectin*	5′-GCACTGGCAAGTTCTACTGCAA-3′	5′-GTAGGTGAAGAGAACGGCCTTGT-3′
*Ap2*	5′-CACCGAGATTTCCTTCAAACT-3′	5′-GCCATCTAGGGTTATGATGC-3′
*βActin*	5′-ATGGCTGGGGTGTTGAAGGT-3′	5′-ATCTGGCACCACACCTTCTACAA-3′
*Cd11c*	5′-GTGCCCATCAGTTCCTTACA-3′	5′-GAGA AGA ACTGTGGAGCTGAC-3′

### Western blot analysis

Protein levels were determined from whole tibialis anterior and gastrocnemius hindlimb skeletal muscles harvested from young (2 months) and old (18 months) WT mice. Protein extracts were harvested in RIPA buffer (Cell Signaling Technology, Danvers, MA, USA), supplemented with protease inhibitors 1 mM PMSF + 1× Protease inhibitor cocktail (Cell Signaling Technology, Danvers, MA, USA). Protein concentration was assayed with bicinchoninic acid assay protein assay reagent (Thermo Fisher Scientific). Equal amounts of protein were fractioned by SDS-PAGE (Mini-Protean Gel, Bio-Rad, CA, USA) and transferred onto a PVDF membrane (Bio-Rad, CA, USA). To perform immunoblotting, membranes were blocked for one hour with 5% non-fat dry milk, and then incubated overnight at 4 °C with anti-FGF2 (sc-79, Santa Cruz, CA, USA), anti-pFGFR1 (ab173305, Abcam, Cambridge, UK), anti-pFGFR3 (ab155960, Abcam, Cambridge, UK), anti-pFGFR4 (PA5-105531, Invitrogen, Carlsbad, CA), anti-pERK (4370S, Cell Signaling Technology, Danvers, MA). Membranes were then incubated with appropriate secondary antibody (Amersham Bioscience, NJ) at room temperature for 1 h. Blots were developed with Supersignal West Dura Extended Duration Substrate (Thermo Scientific, Waltham, MA). Finally, blots were re-probed with Actin antibody (sc-1615, Santa Cruz, CA) or anti-ERK (9102S, Cell Signaling Technology, Danvers, MA). Quantification by densitometric analysis of digitized autoradiograms with NIH Image J software was performed (Supplementary Figures).

### Statistical analysis

Methods utilized were previously published^36,37^. Data are presented as means ± SE. The *t* test, one-way ANOVA, or two-way ANOVA followed by least significant difference (LSD) for post hoc multiple comparisons were used. Differences were considered significant at p values < 0.05.

## Supplementary Information

Supplementary Figures.

## References

[1] Santilli V, Bernetti A, Mangone M, Paoloni M (2014). Clinical definition of sarcopenia. Clin. Cases Miner. Bone Metab..

[2] Khosla S, Bellido TM, Drezner MK, Gordon CM, Harris TB, Kiel DP, Kream BE, LeBoff MS, Lian JB, Peterson CA, Rosen C, Williams JB, Winer KK, Sherman SS (2011). Forum on aging and skeletal health: Summary of the proceedings of an ASBMR workshop. J. Bone Miner. Res..

[3] DiGirolamo DJ, Kiel DP, Esser KA (2013). Bone and skeletal muscle: Neighbors with close ties. J. Bone Miner. Res..

[4] Fielding RA (2011). Sarcopenia: An undiagnosed condition in older adults. Current consensus definition: Prevalence, etiology, and consequences. International working group on sarcopenia. J. Am. Med. Dir. Assoc..

[5] Abreu EL, Stern M, Brotto M (2012). Bone–muscle interactions: ASBMR topical meeting. IBMS BoneKey.

[6] Bonewald LF (2013). Forum on bone and skeletal muscle interactions: Summary of the proceedings of an ASBMR workshop. J. Bone Miner. Res..

[7] Carlson ME, Silva HS, Conboy IM (2008). Aging of signal transduction pathways, and pathology. Exp. Cell Res..

[8] Lefaucher JP, Sebille A (1995). Muscle regeneration following injury can be modified in vivo by immune neutralization of basic fibroblast growth factor, transforming growth factor beta 1 or insulin-like growth factor I. J. Neuroimmunol..

[9] Lefaucher JP, Sebille A (1995). Basic fibroblast growth factor promotes in vivo muscle regeneration in murine muscular dystrophy. Neurosci. Lett..

[10] Hamrick MW, McNeil PL, Patterson SL (2010). Role of muscle-derived growth factors in bone formation. J. Musculoskelet. Neuronal. Interact..

[11] Chakkalakal JV, Jones KM, Basson A, Brack AS (2012). The aged niche disrupts muscle stem cell quiescence. Nature.

[12] Kastner S, Elias MC, Rivera AJ, Yablonka-Reuveni Z (2000). Gene expression patterns of fibroblast growth factors and their receptors during myogenesis of rat satellite cells. J. Histochem. Cytochem..

[13] Fiore F, Sebille A, Birnbaum D (2000). Skeletal muscle regeneration is not impaired in FGF6^−^^/^^−^ mice. Biochem. Biophys. Res. Commun..

[14] Staiger H, Keuper M, Berti L, de Angelis MH, Haring HU (2017). Fibroblast growth factor 21 metabolic role in mice and men. Endocr. Rev..

[15] Montero A (2000). Disruption of the fibroblast growth factor-2 gene results in decreased bone mass and bone formation. J. Clin. Investig..

[16] Xiao L (2010). Disruption of the Fgf2 gene activates the adipogenic and suppresses the osteogenic program in mesenchymal marrow stromal stem cells. Bone.

[17] Moyers JS (2007). Molecular determinants of FGF-21 activity—Synergy and cross-talk with PPARg signaling. J. Cell. Physiol..

[18] Zhou M (1998). Fibroblast growth factor 2 control of vascular tone. Nat. Med..

[19] Kronenberg, M. *et al*. Adipocyte directed expression of GFP in transgenic mice. *J. Bone. Miner. Res*. **19**(Suppl 1) (2004).

[20] Hampton TG (2011). Gait disturbances in dystrophic hampsters. J. Biomed. Biotechnol..

[21] Coffin JD, Homer-Bouthiette C, Hurley MM (2018). Fibroblast growth factor 2 and its receptors in bone biology and disease. J. Endocr. Soc..

[22] Wooley C, Xing S, Burgess RW, Cox GA, Seburn KL (2009). Age, experience and genetic background influence treadmill walking in mice. Physiol. Behav..

[23] Loring SH, Mead J, Waggener TB (1990). Determinants of breathing frequency during walking. Respir. Physiol..

[24] Organ JM (2016). Reduced skeletal muscle function is associated with decreased fiber cross-sectional area in the Cy/+ rat model of progressive kidney disease. Nephrol. Dial. Transpl..

[25] Song MY, Ruts E, Kim J, Janumala I, Heymsfield S, Gallagher D (2004). Sarcopenia and increased adipose tissue infiltration of muscle in elderly African American women. Am. J. Clin. Nutr..

[26] Cree MG (2004). Intramuscular and liver triglycerides are increased in the elderly. J. Clin. Endocrinol. Metab..

[27] Ryall JG, Schertzer JD, Lynch GS (2008). Cellular and molecular mechanisms underlying age-related skeletal muscle wasting and weakness. Biogerontology.

[28] Yoon JH (2012). Secretomics for skeletal muscle cells: A discovery of novel regulators. Adv. Biol. Regul..

[29] Neuhaus P (2003). Reduced mobility of fibroblast growth factor (FGF)-deficient myoblasts might contribute to dystrophic changes in the musculature of FGF2/FGF6/mdx triple-mutant mice. Mol. Cell. Biol..

[30] Armand AS, Laziz I, Canoine C (2006). FGF6 in myogenesis. Biochim. Biophys. Acta.

[31] Izumiya Y, Bina HA, Ouchi N, Akasaki Y, Kharitonenkov A, Walsh K (2008). FGF21 is an Akt-regulated myokine. FEBS Lett..

[32] Itoh N (2014). FGF21 as a hepatokine, adipokine, and myokine in metabolism and diseases. Front. Endocrinol. (Lausanne).

[33] Phelps M, Stuelsatz P, Yablonka-ReuvenI Z (2016). Expression profile and overexpression outcome indicate a role for β-Klotho in skeletal muscle fibro/adipogenesis. FEBS J..

[34] Flanagan-Steet H, Hannon K, McAvoy MJ, Hullinger R, Olwin BB (2000). Loss of FGF receptor 1 signaling reduces skeletal muscle mass and disrupts myofiber organization in the developing limb. Dev. Biol..

[35] Zhao P (2006). Fgfr4 is required for effective muscle regeneration in vivo: Delineation of a MyoD-Tead2-Fgfr4 transcriptional pathway. J. Biol. Chem..

[36] Burt PM, Xiao L, Doetschman T, Hurley MM (2019). Ablation of low-molecular-weight FGF2 isoform accelerates murine osteoarthritis while loss of high-molecular-weight FGF2 isoforms offers protection. J. Cell Physiol..

[37] Meo Burt P, Xiao L, Hurley MM (2018). FGF23 regulates Wnt/β-catenin signaling-mediated osteoarthritis in mice overexpressing high molecular weight FGF2. Endocrinology.

