# Supplementary material for: Gait disturbances and muscle dysfunction in fibroblast growth factor 2 knockout mice
Source: Sci Rep. 2021 May 26;11:11005. doi: 10.1038/s41598-021-90565-0 (PMC8154953; doi:10.1038/s41598-021-90565-0)
Supplement: Supplementary file 1 — Supplementary Figures. [file 41598_2021_90565_MOESM1_ESM.pdf]

Gait Disturbances and Muscle Dysfunction in Fibroblast Growth Factor 2 Knockout Mice

Homer-Bouthiette, C., Xiao, L., & Hurley, M. M.

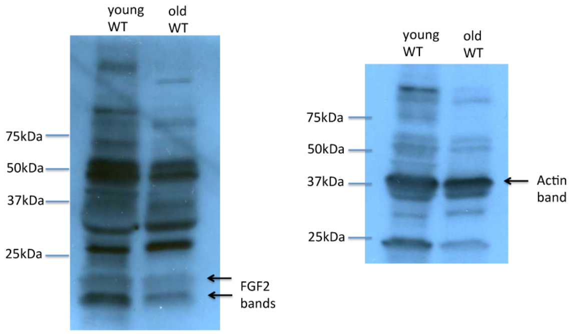

Supplemental Figure 1. Entire uncut Western blots in Figure 3b.

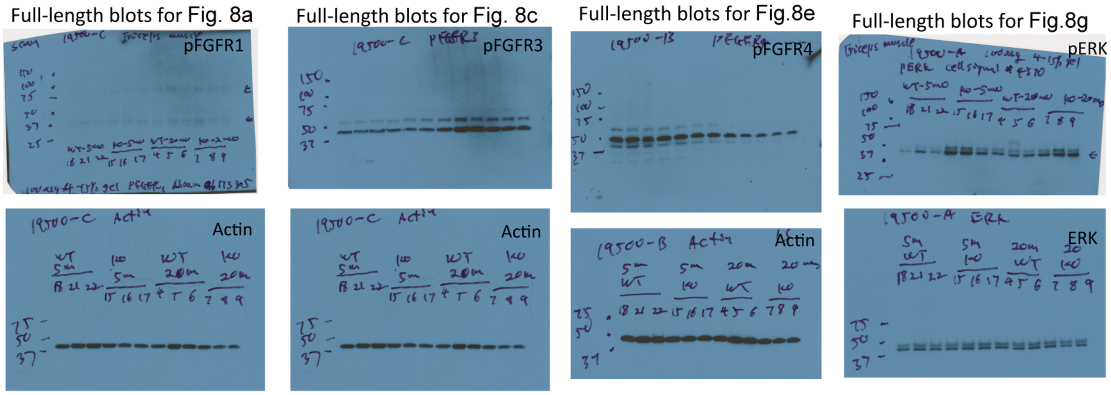

Supplemental Figure 2. Entire uncut Western blots in Figure 8a,c,e,g.
